# Receptor tyrosine kinase amplified gastric cancer: Clinicopathologic characteristics and proposed screening algorithm

**DOI:** 10.18632/oncotarget.12291

**Published:** 2016-09-27

**Authors:** Cheol Keun Park, Ji Soo Park, Hyo Song Kim, Sun Young Rha, Woo Jin Hyung, Jae-Ho Cheong, Sung Hoon Noh, Sang Kil Lee, Yong Chan Lee, Yong-min Huh, Hyunki Kim

**Affiliations:** ^1^ Department of Pathology, Yonsei University College of Medicine, Seoul, Republic of Korea; ^2^ Division of Medical Oncology, Yonsei Cancer Center, Department of Internal Medicine, Yonsei University College of Medicine, Seoul, Republic of Korea; ^3^ Department of Surgery, Yonsei University College of Medicine, Seoul, Republic of Korea; ^4^ Division of Gastroenterology, Department of Internal Medicine, Yonsei University College of Medicine, Seoul, Republic of Korea; ^5^ YUMS-KRIBB Medical Convergence Research Institute, Department of Radiology, Yonsei University College of Medicine, Seoul, Republic of Korea

**Keywords:** gastric cancer, receptor tyrosine kinase, amplification, screening algorithm

## Abstract

Although targeted therapy for receptor tyrosine kinases (RTKs) of advanced gastric cancers (AGCs) has been in the spotlight, guidelines for the identification of RTK-amplified gastric cancers (RA-GCs) have not been established. In this study, we investigate clinicopathologic characteristics of RA-GCs and propose a screening algorithm for their identification. We performed immunohistochemistry (IHC) for MLH1, MSH2, PMS2, MSH6, key RTKs (EGFR, HER2, MET), and p53, *in situ* hybridization for Epstein-Barr virus encoding RNA, and silver *in situ* hybridization (SISH) for *EGFR*, *HER2*, and *MET* using tissue microarrays of 993 AGCs. On IHC, 157 (15.8%) 61, (6.15%), and 85 (8.56%) out of 993 cases scored 2+ or 3+ for EGFR, HER2, and MET, respectively. On SISH, 31.2% (49/157), 80.3% (49/61), and 30.6% (26/85) of 2+ or 3+ cases on IHC showed amplification of the corresponding genes. Of the 993 cases, 104 were classified as RA-GCs. RA-GC status correlated with older age (*P* < 0.001), differentiated histology (*P* = 0.001), intestinal or mixed type by Lauren classification (*P* < 0.001), lymphovascular invasion (*P* = 0.026), and mutant-pattern of p53 (*P* < 0.001). The cases were divided into four subgroups using two classification systems, *putative molecular classification* and *histologic-molecular classification*, based on Lauren classification, IHC, and SISH results. The *histologic-molecular classification* showed higher sensitivity for identification of RA-GCs and predicted patient prognosis better than the *putative molecular classification*. In conclusion, RA-GCs show unique clinicopathologic features. The proposed algorithm based on *histologic-molecular classification* can be applied to select candidates for genetic examination and targeted therapy.

## INTRODUCTION

Gastric cancer (GC) is one of the most common cancers in the world, especially in East Asian countries such as Korea and Japan [1]. Surgery with pre- or postoperative chemotherapy has been performed as a treatment option for advanced gastric cancers (AGCs). However, the majority of patients show poor prognosis and there are unmet clinical needs for this dismal disease [2, 3]. In this regard, targeted therapy that interferes with molecules associated with oncogenesis or disease progression represents a promising solution. In human patients with epidermal growth factor receptor 2 (HER2)-overexpressing gastric cancer, combination treatment with trastuzumab, anti-HER2 antibody, and chemotherapy with cisplatin and fluoropyrimidine-based regimens resulted in longer overall survival than chemotherapy alone [4]. Since this study, several clinical trials that targeted other receptor tyrosine kinases (RTKs) have been performed [5–8]. However, the majority of trials encountered difficult situations due to the rarity of the candidate population. Cases showing amplification of RTK genes only account for a small proportion of total GCs: 5-10% with *epidermal growth factor receptor* (*EGFR*), 6-17% with *HER2*, and 6-12% with *MET* gene amplification [9–14].

According to The Cancer Genome Atlas (TCGA) research, GCs can be divided into four molecular subgroups: Epstein-Barr virus (EBV) positive, microsatellite instability (MSI), genome stable (GS), and chromosomal instability (CIN) [14]. Interestingly, the majority of GCs that overexpress RTKs belong to the CIN group, and GCs in the CIN group show unique characteristics of a higher rate of p53 mutation and mutual exclusiveness in the amplification of RTK genes [14]. With increasing interest in targeted therapy for GCs, it has become more important to identify cases showing amplification of the target genes associated with the specific therapy. Furthermore, considering the rarity of the candidate patients, it is mandatory to develop an adequate and robust platform that is clinically practicable. However, to our best knowledge, algorithms or guidelines to identify RTK-amplified gastric cancers (RA-GCs) and the CIN subgroup have not been established. In this study, we performed comparative analyses using immunohistochemistry (IHC) and *in situ* hybridization, which are clinically feasible assay platforms in terms of speed and cost effectiveness, to investigate clinicopathologic characteristics of RA-GCs. On the basis of these results, we propose a screening algorithm for the identification of RA-GCs.

## RESULTS

### Immunohistochemical profile of AGCs

Expression of mismatch repair (MMR) gene related proteins was evaluated in 990 cases. MMR-deficient GCs were found in 114 cases (11.5%). Simultaneous loss of expression of MLH1 and PMS2 was observed in 101 cases and co-loss of MSH2 and MSH6 was observed in 13 cases.

**Figure 1:**
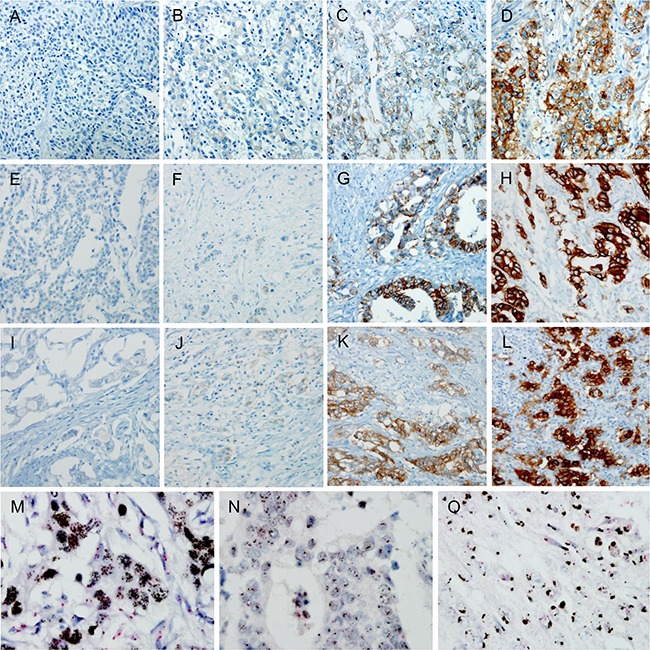
Immunohistochemical (IHC) staining and silver *in situ* hybridization (SISH) for EGFR, HER2, and MET Representative microphotographs of negative, 1+, 2+, and 3+ cases for EGFR, HER2, and MET (A-D, EGFR; E-H, HER2; I-L, MET) with original magnification ×200. Representative cases of gene amplification for *EGFR, HER2,* and *MET* SISH (M-O) with original magnification ×400.

All 993 cases were evaluated for expression of EGFR, HER2, MET, and p53. The number of cases scored as 0, 1+, 2+, and 3+ respectively was 524 (52.8%), 312 (31.4%), 119 (12.0%), and 38 (3.8%) for EGFR (Figure 1A to 1D); 846 (85.4%), 86 (8.7%), 29 (2.9%), and 32 (3.2%) for HER2 (Figure 1E to 1H); and 726 (73.1%), 182 (18.3%), 67 (6.7%), and 18 (1.8%) for MET (Figure 1I to 1L). Thus, 157 (15.8%), 61 (6.1%), and 85 (8.5%) cases were classified as positive for EGFR, HER2, and MET, respectively. In p53 IHC, 371 (37.4%) and 622 (62.6%) cases were classified as wild-pattern and mutant-pattern, respectively.

Clinicopathologic characteristics according EGFR, HER2 and MET IHC results are summarized in Table 1. EGFR positivity and HER2 positivity were more frequent in older patients (*P* = 0.003, and 0.013, respectively) and MET expression showed a similar tendency (*P= 0*.069). Positivity for each of the three RTKs was significant in AGCs with differentiated histology (*P* < 0.001 for EGFR and HER2; *P* = 0.032 for MET) and intestinal or mixed type by Lauren classification (*P* < 0.001 for all). Additionally, EGFR positivity was associated with male sex (*P* = 0.036), lower third location (*P* = 0.004), EBV encoding RNA *in situ* hybridization (EBER-ISH) negativity (*P* = 0.001), and MMR deficiency (*P* < 0.001). HER2 positivity was frequently observed in cases with p53 mutant-pattern (*P* = 0.009). MET positivity was associated with larger tumor size (> 5 cm, *P* = 0.041), presence of lymphovascular invasion (LVI, *P* = 0.025), lymph node metastasis (LNM, *P* = 0.030), and MMR deficiency (*P* < 0.001).

**Table 1:** Clinicopathologic characteristics of advanced gastric cancers according to the expression status of EGFR, HER2, and MET

Category	Variables	No. of cases(*n* = 993)	EGFR					HER2					c-MET				
			Positive (%)		Negative (%)		*P-*value	Positive (%)		Negative (%)		*P-*value	Positive (%)		Negative (%)		*P-*value
			(*n* = 157)		(*n* = 836)			(*n* = 61)		(*n* = 932)			(*n* = 85)		(*n* = 908)		
Age (years)			58.5 ± 10.7		55.7 ± 12.8		0.003	60.0 ± 10.7		55.9 ± 12.6		0.013	58.5 ± 10.7		55.9 ± 12.7		0.069
Sex	Male	647	114	(72.6)	533	(63.8)	0.036	46	(75.4)	601	(64.5)	0.096	61	(71.8)	586	(64.5)	0.192
	Female	346	43	(27.4)	303	(36.2)		15	(24.6)	331	(35.5)		24	(28.2)	332	(35.5)	
Location	Lower third	552	104	(66.2)	448	(53.6)	0.004	38	(62.3)	514	(55.2)	0.291	55	(64.7)	497	(54.7)	0.087
	Upper and mid-third	441	53	(33.8)	388	(46.4)		23	(37.7)	418	(44.8)		30	(35.3)	411	(45.3)	
Size	≤ 5 cm	495	70	(44.6)	425	(50.8)	0.164	31	(50.8)	464	(49.8)	0.896	33	(38.8)	462	(50.9)	0.041
	> 5 cm	498	87	(55.4)	411	(49.2)		30	(49.2)	468	(50.2)		52	(61.2)	446	(49.1)	
Differentiation	Differentiated	281	70	(44.6)	211	(25.2)	< 0.001	30	(49.2)	251	(26.9)	< 0.001	33	(38.8)	248	(27.3)	0.032
	Undifferentiated	712	87	(55.4)	625	(74.8)		31	(50.8)	681	(73.1)		52	(61.2)	660	(72.7)	
Lauren classification	Intestinal or mixed	518	125	(79.6)	393	(47.0)	< 0.001	47	(77.0)	471	(50.5)	< 0.001	73	(85.9)	445	(49.0)	< 0.001
	Diffuse	475	32	(20.4)	443	(53.0)		14	(23.0)	461	(49.5)		12	(14.1)	463	(51.0)	
LVI	Absent	704	102	(65.0)	602	(72.0)	0.085	40	(65.6)	664	(71.2)	0.383	51	(60.0)	653	(71.9)	0.025
	Present	289	55	(35.0)	234	(28.0)		21	(34.4)	268	(28.8)		34	(40.0)	255	(28.1)	
LNM	Absent	275	44	(28.0)	231	(27.6)	0.919	15	(24.6)	260	(27.9)	0.576	15	(17.6)	260	(28.6)	0.030
	Present	718	113	(72.0)	605	(72.4)		46	(75.4)	672	(72.1)		70	(82.4)	648	(71.4)	
Pathologic T stage	T2	163	26	(16.6)	137	(16.4)	0.012	9	(14.8)	154	(16.5)	0.385	11	(12.9)	152	(16.7)	0.208
	T3	358	72	(45.9)	286	(34.2)		27	(44.3)	331	(35.5)		38	(44.7)	320	(35.2)	
	T4	472	59	(37.6)	413	(49.4)		25	(41.0)	447	(48.0)		36	(42.4)	436	(48.0)	
p53 IHC	Wild-type pattern	371	63	(40.1)	308	(36.8)	0.472	13	(21.3)	358	(38.4)	0.009	30	(35.3)	341	(37.6)	0.726
	Mutant pattern	622	94	(59.9)	528	(63.2)		48	(78.7)	574	(61.6)		55	(64.7)	567	(62.4)	
EBER-ISH*	Negative	910	150	(99.3)	760	(92.7)	0.001	60	(98.4)	850	(93.4)	0.170	78	(94.0)	832	(93.7)	0.919
	Positive	61	1	(0.7)	60	(7.3)		1	(1.6)	60	(6.6)		5	(6.0)	56	(6.3)	
MMR protein IHC**	MMR-proficient	876	112	(71.8)	764	(91.6)	< 0.001	56	(91.8)	820	(88.3)	0.402	63	(75.0)	813	(89.7)	< 0.001
	MMR-deficient	114	44	(28.2)	70	(8.4)		5	(8.2)	109	(11.7)		21	(52.0)	93	(10.3)	
Overall stage	II	95	16	(10.2)	79	(9.4)	0.690	5	(8.2)	90	(9.7)	0.931	6	(7.1)	89	(9.8)	0.094
	III	307	44	(28.0)	263	(31.5)		19	(31.1)	288	(30.9)		19	(22.4)	288	(31.7)	
	IV	591	97	(61.8)	494	(59.1)		37	(60.7)	554	(59.4)		60	(70.6)	531	(58.5)	

LVI: lymphovascular invasion; LNM: lymph node metastasis; IHC: immunohistochemistry; EBER-ISH: Epstein-Barr virus encoding RNA *in situ* hybridization; MMR protein: mismatch repair gene related protein

* Evaluated in 971 cases

** Evaluated in 990 cases

### RTK gene amplification and EBV profile of AGCs

Among 2+ or 3+ cases for each RTK on IHC, 31.2% (49/157 cases), 80.3% (49/61 cases), and 30.6% (26/85 cases) were revealed the amplifications of *EGFR*, *HER2*, and *MET* gene, respectively (Figure 1M to 1Q) on silver *in situ* hybridization (SISH). Co-amplification of RTK genes were observed in eighteen cases with following combinations: 7 cases with co-amplification of *EGFR* and *HER2*, 6 cases with co-amplification of *EGFR* and *MET,* 4 cases with co-amplification of *HER2* and *MET*, and one case with co-amplification of all three RTK genes. Evaluation of EBER-ISH was available in 971 out of 993 cases and 61 (6.3%) cases were EBV-positive.

### Clinicopathologic characteristics of RA-GCs

Of 993 cases, 104 (10.5%) were identified as RA-GCs, including 18 cases showing co-amplification. RA-GCs showed unique clinicopathologic characteristics (Table 2); they were associated with older age (*P* < 0.001), differentiated histology (*P* = 0.001), intestinal or mixed type by Lauren classification (*P* < 0.001), presence of LVI (*P* = 0.026), and p53 mutant-pattern (*P* < 0.001). Among the 104 RA-GCs, 18 co-amplified AGCs showed no significant differences in clinicopathologic features compared to 86 RA-GCs without co-amplification (Supplementary Table S1).

**Table 2:** Clinicopathologic characteristics of advanced gastric cancers according to gene amplification status of three receptor tyrosine kinases (RTKs)

Category	Variables	No. of cases (*n* = 993)	RTK gene amplification*				*P-*value
			Positive (%)		Negative (%)		
			(*n* = 104)		(*n* = 889)		
Age (years)			60.3 ± 9.7		55.6 ± 12.7		< 0.001
Sex	Male	647	76	(73.1)	571	(64.2)	0.073
	Female	346	28	(26.9)	319	(35.8)	
Location	Lower third	552	66	(63.5)	486	(54.7)	0.088
	Upper and mid-third	441	38	(36.5)	403	(45.3)	
Size	≤ 5 cm	495	52	(50.0)	443	(49.8)	0.974
	> 5 cm	498	52	(50.0)	446	(50.2)	
Histology	Differentiated	281	44	(42.3)	237	(26.7)	0.001
	Undifferentiated	712	60	(57.7)	652	(73.3)	
Lauren classification	Intestinal or mixed	538	90	(86.5)	448	(50.4)	< 0.001
	Diffuse	455	14	(13.5)	441	(49.6)	
LVI	Absent	704	64	(61.5)	640	(72.0)	0.026
	Present	289	40	(38.5)	249	(28.0)	
LNM	Absent	275	26	(25.0)	249	(28.0)	0.516
	Present	718	78	(75.0)	640	(72.0)	
Pathologic T stage	T2	163	19	(18.3)	144	(16.2)	0.140
	T3	358	45	(43.3)	313	(35.2)	
	T4	472	40	(38.5)	432	(48.6)	
p53 IHC	Wild-type pattern	371	22	(21.2)	349	(39.3)	< 0.001
	Mutant pattern	622	82	(78.8)	540	(60.7)	
EBER-ISH**	Negative	910	100	(98.0)	810	(93.2)	0.057
	Positive	61	2	(2.0)	59	(6.8)	
MMR protein IHC***	MMR-proficient	876	96	(93.2)	780	(87.9)	0.113
	MMR-deficient	114	7	(6.8)	107	(12.1)	
Overall stage	II	95	13	(12.5)	82	(9.2)	0.210
	III	307	25	(24.0)	282	(31.7)	
	IV	591	66	(63.5)	525	(59.1)	

LVI: lymphovascular invasion; LNM: lymph node metastasis; IHC: immunohistochemistry; EBV-ISH Epstein-Barr virus encoding RNA *in situ* hybridization; MMR protein: mismatch repair gene related protein

* Defined as amplification of any of EGFR, HER2 or MET in SISH

** Evaluated in 971 cases

*** Evaluated in 990 cases

### Sensitivity for identifying RA-GCs according to classification system

We divided all AGC cases into four subgroups based on two classification systems: *putative molecular* and *histologic-molecular classification.* According to the *putative molecular classification*, the 993 AGCs were composed of the following subgroups: 61 (6.1%) EBV-positive GCs, 114 (11.5%) MMR-deficient GCs, 253 (25.5%) putative GS (pGS) GCs, and 565 (56.9%) putative CIN (pCIN) GCs. The *histologic-molecular classification* divided the AGCs into the following four subgroups: 61 (6.1%) EBV-positive GCs, 114 (11.5%) MMR-deficient GCs, 143 (14.4%) diffuse-putative GS (D-pGS) GCs, and 675 (68.0%) intestinal-putative CIN (I-pCIN) GCs.

Next, we compared *putative molecular classification* and *histologic-molecular classification* to investigate which system was superior for screening out RA-GCs. A correlation between RTK IHC positivity and I-pCIN group of *histologic-molecular classification* was significant (*P* < 0.001; Table 3). However, pCIN group of *putative molecular classification was not.* Both pCIN and I-pCIN groups correlated well with RTK amplification (*P* = 0.007 and < 0.001, respectively, Table 3). I-pCIN group showed higher sensitivity (87.5%) for identifying RA-GCs than pCIN group of *putative molecular classification* (74.0%) (Table 3). Therefore, when mining RTK amplified cases, the *histologic-molecular classification* was superior to *putative molecular classification* in this study. However, both pCIN and I-pCIN groups showed low specificity to find EGFR, HER2, or MET amplified-GCs (45.1% and 34.3%, respectively, Table 3).

**Table 3:** RTK expression and amplification status according to classification systems

Classification system	Subgroup	No. of cases(*n* = 993)	RTK IHC					RTK SISH				
			Positive (%)		Negative (%)		*P-*value	Amplified (%)		Not amplified (%)		*P-*value
			(*n* = 230)		(*n* = 763)			(*n* = 104)		(*n* = 889)		
*Putative molecular*	EBV-postive	61	5	(2.2)	58	(7.3)	0.003^†^	2	(1.9)	59	(6.6)	0.009^†^
	MMR-deficient	114	51	(22.2)	63	(8.3)		7	(6.7)	107	(12.0)	
	pGS	253	47	(20.4)	206	(27.0)	0.208^††^	18	(17.3)	235	(26.4)	0.007^††^
	pCIN	565	127	(55.2)*	438	(57.4)**		77	(74.0)*	488	(54.9)**	
*Histoligic-molecular*	EBV-postive	61	5	(2.2)	58	(7.3)	0.003^†^	2	(1.9)	59	(6.6)	0.009^†^
	MMR-deficient	114	51	(22.2)	63	(8.3)		7	(6.7)	107	(12.0)	
	D-pGS	143	11	(4.8)	132	(17.3)	< 0.001^††^	4	(3.8)	139	(15.6)	< 0.001^††^
	I-pCIN	675	163	(70.9)*	512	(67.1)**		91	(87.5)*	584	(65.7)**	

MMR: mismatch repair gene related; pGS: putative genome stable; pCIN: putative chromosome instability; D-pGS: diffuse-putative genome stable; I-pCIN: intestinal-putative chromosome instability

† *P*-value for the comparison of EBV positive + MMR deficient *vs.* pGS + pCIN or *vs.* D-pGS + I-pCIN

†† *P*-value for the comparison of pGS *vs.* pCIN or D-pGS *vs.* I-pCIN

* Sensitivity of pCIN and I-pCIN subgroup for the detection of RTK IHC positive case or RTK amplified case.

** Specificity of pCIN and I-pCIN subgroup for the detection of RTK IHC positive case or RTK amplified case: 100-(%)^**^.

### Survival analysis

Survival analysis was available for 979 of 993 patients. Kaplan-Meier survival curves showed no significant differences in recurrence-free survival (RFS) and overall survival (OS) depending on RTK expression (Supplementary Figure S1A and S1B for EGFR IHC, C and D for HER2 IHC, and E and F for MET IHC). In addition, the RFS and OS of patients with AGC showing any RTK positivity on IHC did not differ from the remaining groups (Supplementary Figure S1G and S1H). The prognosis of patients with AGC showing amplification of any of the three RTK genes also did not vary significantly according to amplification status (Supplementary Figure S2A and S2B for *EGFR*, C and D for *HER2*, and E and F for *MET*). In addition, there were no significant differences in RFS and OS between RA-GCs and non-RA-GCs (Supplementary Figure S3A and S3B) and no significant findings were observed in survival analysis after the separation of RTK co-amplified cases (Supplementary Figure S3C and S3D).

**Figure 2:**
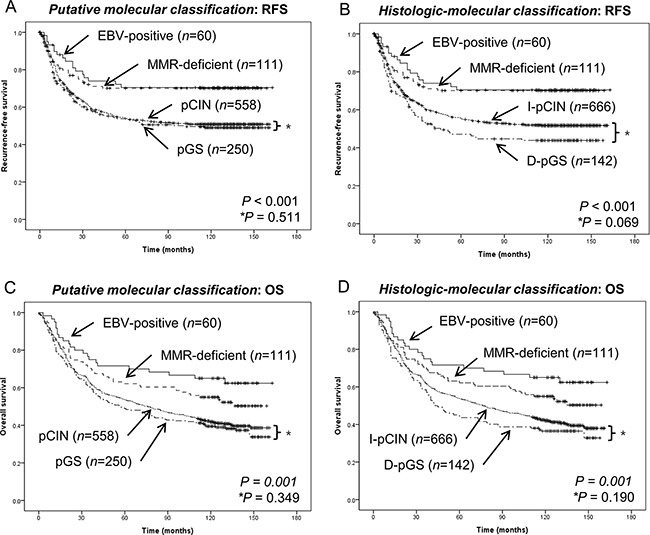
Comparison of recurrence-free survival and overall survival according to the group classification method **A.** Recurrence-free survival according to *putative molecular classification*. EBV-positive and MMR-deficient subgroups showed favorable prognosis. However, pGS and pCIN subgroups showed relatively poor recurrence-free survival. Separate analysis of pGS and pCIN subgroups showed no significant difference between the two subgroups (*P* = 0.511). **B.** Recurrence-free survival according to *histologic-molecular classification*. On separate analysis of D-pGS and I-pCIN subgroups, the I-pCIN subgroup showed a trend toward longer recurrence-free survival (*P* = 0.069). **C.** Overall survival according to the *putative molecular classification*. **D.** Overall survival according to the *histologic-molecular classification*.

In the *putative molecular classification,* EBV-positive and MMR-deficient subgroups showed longer RFS whereas pGS and pCIN subgroups showed relatively shorter RFS (*P* = 0.001); however, there was no significant difference in RFS between pGS and pCIN subgroups (*P* = 0.511, Figure 2A). In contrast, for *histologic-molecular classification,* in addition to significant differences observed in comparison of entire subgroups (*P* = 0.001), the I-pCIN subgroup showed a trend toward longer RFS than the D-pGS subgroup (*P* = 0.069; Figure 2B). In multivariate analysis, D-pGS and I-pCIN subgroups of histologic-molecular classification were worse prognostic factors for RFS (p=0.012, HR 2.07 and p=0.014, HR 1.93, respectively) and OS (p=0.001, HR 2.27 and p=0.002 HR 2.1, respectively) (Table 4).

**Table 4:** Multivariate analysis of the impact of clinicopathologic factors on recurrence/metastasis and survival

Category	Variables	Recurrence/metastasis (*Putative molecular*)		Survival (*Putative molecular*)		Recurrence/metastasis (*Histologic-molecular*)		Survival (*Histologic-molecular*)	
		HR (95% CI)	*P-*value	HR (95% CI)	*P-*value	HR (95% CI)	*P-*value	HR (95% CI)	*P-*value
Sex	Male	1		1		1		1	
	Female	1.028 (0.836-1.263)	0.797	0.973 (0.812-1.166)	0.768	1.028 (0.836-1.264)	0.796	0.972 (0.811-1.164)	0.758
Age (years)	< 60	1		1		1		1	
	≥ 60	1.057 (0.865-1.293)	0.588	1.556 (1.307-1.853)	< 0.001	1.055 (0.862-1.290)	0.605	1.554 (1.306-1.850)	< 0.001
Location	Lower third	1		1		1		1	
	Upper and mid-third	1.075 (0.881-1.311)	0.477	1.259 (1.060-1.495)	0.009	1.079 (0.885-1.316)	0.453	1.264 (1.064-1.501)	0.008
Size	≤ 5 cm	1		1		1		1	
	> 5 cm	1.282 (1.043-1.575)	0.018	1.197 (0.999-1.433)	0.051	1.284 (1.045-1.579)	0.017	1.201 (1.002-1.438)	0.047
Histology	Differentiated	1		1		1		1	
	Undifferentiated	0.830 (0.625-1.103)	0.199	0.831 (0.658-1.051)	0.122	0.834 (0.628-1.107)	0.208	0.832 (0.658-1.052)	0.124
Lauren classification	Intestinal and mixed	1		1		1		1	
	Diffuse	1.361 (1.050-1.763)	0.020	1.232 (0.989-1.534)	0.063	1.332 (1.002-1.770)	0.048	1.203 (0.944-1.534)	0.135
LVI	Absent	1		1		1		1	
	Present	1.513 (1.230-1.860)	< 0.001	1.550 (1.292-1.859)	< 0.001	1.523 (1.240-1.872)	<0.001	1.566 (1.306-1.877)	< 0.001
LNM	Absent	1		1		1		1	
	Present	1.212 (0.755-1.946)	0.427	1.426 (1.001-2.030)	0.049	1.227 (0.764-1.971)	0.394	1.426 (1.001-2.031)	0.050
Pathologic T stage	T2 and T3	1		1		1		1	
	T4	1.678 (1.329-2.119)	<0.001	1.603 (1.312-1.959)	< 0.001	1.669 (1.323-2.107)	<0.001	1.592 (1.303-1.944)	< 0.001
Overall stage	II and III	1		1		1		1	
	IV	2.657 (1.726-4.090)	< 0.001	1.860 (1.345-2.573)	< 0.001	2.644 (1.717-4.072)	<0.001	1.854 (1.340-2.565)	< 0.001
p53 IHC	Wild-type pattern	1		1		1		1	
	Mutant pattern	1.401 (0.780-2.513)	0.259	1.420 (0.886-2.277)	0.146	0.973 (0.708-1.337)	0.865	0.973 (0.750-1.261)	0.834
RTK IHC	Negative	1		1		1		1	
	Positive*	0.930 (0.673-1.286)	0.662	0.822 (0.625-1.083)	0.163	0.930 (0.672-1.285)	0.659	0.817 (0.621-1.075)	0.149
RTK gene amplification	Negative	1		1		1		1	
	Amplified**	1.130 (0.749-1.706)	0.559	1.160 (0.816-1.649)	0.408	1.126 (0.746-1.699)	0.573	1.155 (0.811-1.642)	0.422
Classification system***	EBV-positive	1		1		1		1	
	MMR-deficient	1.290 (0.700-2.378)	0.415	1.573 (0.937-2.641)	0.086	1.298 (0.703-2.397)	0.405	1.593 (0.947-2.677)	0.079
	pGS / D-pGS	2.325 (1.332-4.060)	0.003	2.555 (1.578-4.136)	< 0.001	2.072 (1.172-3.662)	0.012	2.274 (1.378-3.752)	0.001
	pCIN / I-pCIN	1.472 (0.784-2.764)	0.229	1.573 (0.918-2.695)	0.099	1.934 (1.141-3.277)	0.014	2.100 (1.327-3.324)	0.002

HR: hazard ratio; CI: confidence interval; LVI: lymphovascular invasion; LNM: lymph node metastasis; RTK: receptor tyrosine kinase; MMR: mismatch repair gene related; pGS: putative genome stable; pCIN: putative chromosome instability; D-pGS: diffuse-putative genome stable; I-pCIN: intestinal-putative chromosome instability

* Defined as 2+ or 3+ for any of EGFR, HER2, or MET by IHC.

** Defined as amplification of any of EGFR, HER2, or MET by SISH.

*** *Putative molecular classification* was categorized as EBV-positive, MMR-deficient, pGS and pCIN subgroup. *Histologic-molecular classification* was categorized as EBV-positive, MMR-deficient, D-pGS and I-pCIN subgroup.

Overall survival was different among subgroups for both classification systems (*P* = 0.001 for all; Figure 2C and 2D). However, in multivariate analysis, individual subgroups of *putative molecular classification* did not have a prognostic impact on patient survival except for the pGS subgroup whereas the D-pGS and I-pCIN subgroups of *histologic-molecular classification* were revealed as unfavorable prognostic factors for patient survival (Table 4).

### Proposed screening algorithm

**Figure 3:**
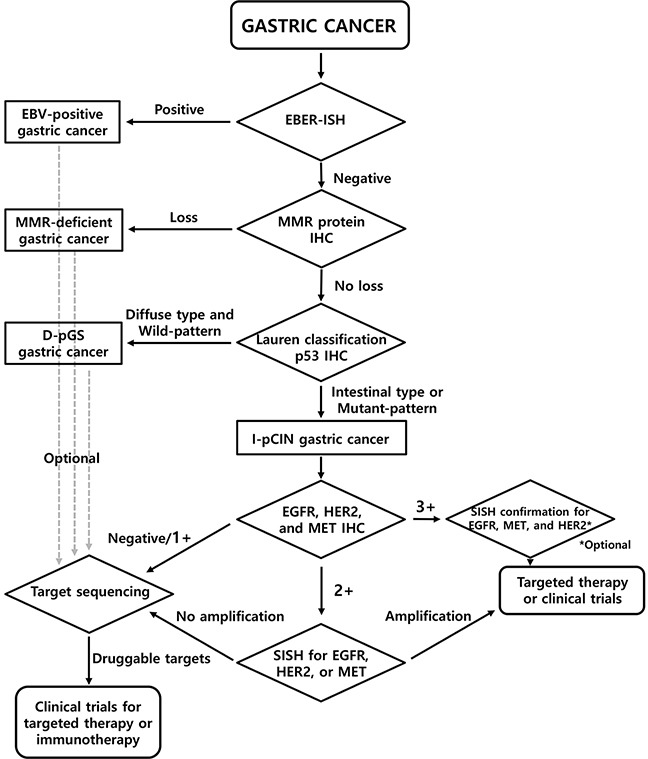
Proposed screening algorithm for the identification of RA-GCs

On comparison of the two classification systems for IHC, *histologic-molecular classification* showed higher sensitivity for identifying RA-GCs than *putative molecular classification*. When considering the significant correlation between RTK IHC positivity and RTK gene amplification (Spearman's correlation coefficient = 0.576, *P* < 0.001), it is expected that *histologic-molecular classification* would identify more RA-GCs than *putative molecular classification*. Moreover, *histologic-molecular classification* can predict patient prognosis much better than *putative molecular classification.* On the basis of these findings, we proposed a screening algorithm for the identification of RA-GCs (Figure 3).

## DISCUSSION

Several studies investigating target genes such as *EGFR*, *MET*, and *fibroblast growth factor receptor* (*FGFR*) have been conducted since the application of trastuzumab in the treatment of HER2-positive gastric cancer patients [11, 15–17]. Based on these studies, several targeted agents that interfere with RTKs have been developed and applied in clinical trials [18–20]. For clinical application and success of clinical trials, it is crucial to select candidates expected to show a response to the targeted agents, in particular, robustly and cost-effectively. The TCGA study [14], which was based on comprehensive molecular analysis, and a study by Cristescu et al. [21], which used transcriptome analysis, categorized GCs into molecular subtypes. RA-GCs were enriched in a subgroup of both of studies: CIN group in the TCGA study and microsatellite stable (MSS)/TP53-positive group in Cristescu et al. [14, 21]. Therefore, these classification approaches can be used to select RTK-amplified candidates. However, several hurdles including quality control, turn-around time, and cost issues can hamper the use of genome- or transcriptome-wide classification in daily clinical practice. To overcome these obstacles, we sought to develop a screening algorithm based on IHC and EBER-ISH methods that are robust, widely used in most pathology labs, and that also can be applied to formalin-fixed, paraffin-embedded materials. In this IHC-based screening strategy, the availability of reliable and robust antibodies is critical. However, we failed to identify reliable antibodies for HER3 and FGFR2, which are other frequently amplified RTKs in GCs. The amplification of these RTKs can be accessed using fluorescence in situ hybridization [22–25], which can be time-consuming and expensive. In this respect, we evaluated the clinicopathologic characteristics of RA-GCs for three RTKs, EGFR, HER, and MET, and sought to develop a screening algorithm for the detection of a subgroup enriched with RA-GCs.

RA-GCs were associated with intestinal or mixed type by Lauren classification, negativity in EBER-ISH, and MMR-proficient and p53 mutant-pattern in IHC; these features are concordant with the findings of a previous genome-wide study [14]. From these findings, we established a *histologic-molecular classification* system using Lauren classification and the results of IHC and *in situ* hybridization. This classification system showed high sensitivity for detecting RA-GCs. Interestingly, overall survival of the subgroups based on *histologic-molecular classification* were similar to those of MSI, MSS/TP53-positive, and MSS/TP53-negative groups classified by Cristescu et al. [21], except for the EBV-positive subgroup of our study and the MSS/EMT group of the report by Cristescu et al. [21].

Although several studies have reported that overexpression or amplification of RTKs is associated with a poor prognosis [11, 15, 16], we could not find any association with prognosis for EGFR, HER2, and MET overexpression/positivity or amplification and did not observe any difference between RA-GCs and non-RA-GCs in this study. The effects of RTK status on patient prognosis are presumed to vary depending on the proportion of EBV-positive or D-pGS patients in the study population. Because we found a negative correlation between EGFR positivity and EBV-positive GCs and a tendency of negative correlation between HER2 positivity and EBV positivity (Table 1), the favorable prognosis of EBV-positive GCs might have confounding effects on the survival analysis of GCs with EGFR and HER2 overexpression. That is, if the proportion of EBV-positive GCs increases in one study population, the EGFR or HER2-overexpressing/positive group might be interpreted as showing a worse prognosis. Inversely, as there is a negative relationship between RTK amplification and the D-pGS subgroup (Table 3), if the proportion of the D-pGS group increases, patients with RTK-amplified GC might show a more favorable prognosis. Thus, in the prognostic evaluation of RA-GCs, the influence of EBV-positive and D-pGS subgroups should be considered.

Most RA-GCs belonged to the I-pCIN subgroup in *histologic-molecular classification*. However, there were 13 cases that belonged to other subgroups: two EBV-positive GCs, seven MMR-deficient GCs, and four D-pGS GCs. These 13 cases consisted of five *EGFR*-amplified and seven *HER2*-amplified GCs, and one case with co-amplification of *EGFR* and *MET*. The presence of these 13 RA-GCs could be a key limitation of our screening algorithm. Although these patients also could be candidates for RTK-targeted therapy, we believe that our classification approach is still valuable. A recent clinical trial using programmed death 1 blockade, pembrolizumab improved outcomes of MMR-deficient colon cancer patients [26]. EBV-positive GCs have shown frequent amplification of the *programmed death ligand 1* (*PDL-1*) gene and overexpression of PDL-1 in tumor cells [14, 27]. Therefore, we speculate that patients with EBV-positive and MMR-deficient GC might be candidates for immunotherapy using immune checkpoint modulators such as pembrolizumab.

In addition to the presence of *EGFR*- and *HER2*-amplified cases in non I-pCIN subgroups, our classification system showed quite low specificity (32.9% for RTK IHC and 34.2% for RTK gene amplification). This may be due to the presence of other RTKs such as HER3, FGFR1, and FGFR2 and downstream molecules such as PIK3CA or PTEN of the RTK pathway [14]. For these RTKs or targetable molecules, an optional target sequencing strategy using next generation sequencing (NGS) could be considered (Figure 3).

Although HER2 3+ expression on IHC has been accepted as a sufficient indicator for trastuzumab-based chemotherapy, the treatment response and clinical outcomes can be affected by the level of *HER2* amplification [28]. Therefore, we included SISH confirmation for HER2 3+ cases as an optional assay in the proposed screening algorithm, which may be informative for predicting response. For EGFR or MET, the concordance between 3+ expression on IHC and gene amplification and the copy number effect on the prediction of response in targeted therapy remain to be elucidated. Therefore, SISH confirmation for *MET* or *EGFR* 3+ cases was included as an essential step in the algorithm. Cases with no overexpression or amplification of EGFR, HER2, and MET in IHC or SISH can be applied to target sequencing to find alterations in other RTKs or RTK-related genes. In this respect, the proposed screening algorithm can provide a stepwise approach for the identification of RTK gene amplification in a clinically feasible and cost-effective way by screening with IHC, validation of equivocal cases by IHC via *in situ* hybridization, and then target sequencing for negative cases.

Despite the advantages mentioned above, this study has several limitations. First, IHC and SISH of RTKs were performed using tissue microarrays (TMAs), raising the possibility of heterogeneity of RTKs in IHC and SISH [29]. An up to 30% rate of tumor heterogeneity on HER2 IHC has been reported in GCs [30, 31]. At the time of TMA construction, we selected the most representative area of each case and used two relatively large cores (3 mm) to reduce the limitations related to tumor heterogeneity. Despite this effort, tumor heterogeneity might still influence our results for the three RTKs.

Second, genetic studies for *TP53* mutation and MSI status were not performed, so there might be differences between IHC results of p53 and MMR proteins and the results of genetic studies. However, several studies have validated the high accordance rate between IHC and genetic study results for p53 and MMR proteins. In ovarian cancer, p53 IHC showed significant correlation with *TP53* mutation status in cases showing complete loss of nuclear expression or strong and diffuse nuclear expression in more than 60% of tumor cells [32]. In colon cancer, the concordance rate between IHC of MMR proteins and PCR-based analysis has been reported to be as high as 98.6% [33–36]. Thus, according to the National Comprehensive Cancer Network guideline for colon cancer, IHC for MMR proteins has been introduced as an alternative method for detecting MSI-H-type colon cancer [37]. In GC, several studies have demonstrated high concordance between these two methods [21, 38, 39]. We also recently validated the high sensitivity and specificity of the IHC approach for MSI detection in GC [40]. Thus, we believe that IHC for p53 and MMR proteins can be a cost-effective modality for the prediction of genetic status in GC.

In conclusion, RA-GCs showed unique clinico pathologic characteristics: occurrence in older patients, differentiated histology, intestinal or mixed type by Lauren classification, presence of LVI, and p53 mutant-pattern. *Histologic-molecular classification* based on histologic type, IHC, and EBER-ISH profiles showed 87.5% sensitivity for the identification of RA-GCs. According to the proposed screening algorithm based on *histologic-molecular classification*, RA-GCs can be identified in a cost-effective way. The positively screened cases might be candidates for clinical trials for the development of new targeted therapies; the negatively screened ones can be the candidates for the investigation of novel RTK genes through additional studies such as NGS.

## MATERIALS AND METHODS

### Case selection and tissue microarray construction

Tissue specimens from 993 consecutive AGC patients who underwent radical gastrectomy from 2000 to 2003 at Severance Hospital were used in this study. Cases that were treated with neoadjuvant chemotherapy were excluded. This study was approved by the Institutional Review Board of Severance Hospital, Seoul, Republic of Korea (4-2015-0616). Various clinicopathologic factors, including age at operation, sex, tumor size and location, and clinical follow-up data were obtained by medical record review. RFS time was calculated from the date of curative resection to the date of the first locoregional or systemic recurrence, or death without any type of relapse. OS time was calculated from the date of curative resection to the date of the last follow-up or death from any cause.

Several pathologic factors, including tumor histology, tumor type by Lauren classification, LVI, perineural invasion, and pathologic TNM staging according to the 7^th^ American Joint Committee on Cancer criteria were obtained from the slide review by two individual pathologists (C. K. Park and H. Kim). Tumor histology was classified as differentiated and undifferentiated based on Japanese gastric cancer treatment guidelines 2010 [41]. Two cores were extracted from a representative tumor area of each case for TMA construction as previously described [40, 42]. For the evaluation of gene amplification via SISH, separate TMAs were constructed for cases showing 2+ or 3+ EGFR, HER2 and MET expression on IHC.

### Immunohistochemistry and evaluation

Four-micrometer tissue sections from TMA recipient blocks were used for IHC. IHC was performed using the Ventana Discovery XT automated staining system (Ventana Medical Systems, Inc., Tucson, AZ, USA) with primary antibodies (Table 5) as previously described [40, 42].

**Table 5:** Antibodies used for immunohistochemical staining

Antibody	Source	Clone	Dilution
MLH1	Roche, Basel, Switzerland	M1	Ready to use
MSH2	Roche, Basel, Switzerland	G219-1129	Ready to use
MSH6	Cell Marque, Rocklin, CA, USA	44	1:100
PMS2	Cell Marque, Rocklin, CA, USA	MRQ28	1:40
p53	Novocastra, Newcastle, UK	DO7	1:300
EGFR	Cell signaling, Danvers, MA, USA	Tyr992	1:200
HER2	Roche, Basel, Switzerland	4B5	Ready to use
c-MET	Roche, Basel, Switzerland	SP44	Ready to use

MLH1: MutL homolog 1; MSH2: MutS protein homolog 2; MSH6: MutS homolog 6; PMS2: Postmeiotic segregation increased 2; EGFR: epidermal growth factor receptor; HER2: human epidermal growth factor receptor-2

Immunostained slides were evaluated by two individual pathologists (C. K. Park and H. Kim) and results were interpreted as follows: cases showing complete loss of MLH1/ PMS2 or MSH2/MSH6 in tumor cells were regarded as MMR-deficient and all other cases were considered MMR-proficient. For p53, cases were classified as p53 mutant-pattern (cases with complete loss of expression or strong nuclear expression in more than 50% of tumor cells) and p53 wild-pattern (all other cases). Expression of EGFR, HER2, and MET was scored according to Hofmann's criteria [30]. Cases scored as 2+ or 3+ were considered positive.

### *In situ* hybridization and evaluation

SISH was performed on cases scored as 2+ or 3+ in IHC for EGFR, HER2, and MET using the Ventana Benchmark XT (Ventana Medical Systems, Inc.) as previously described [12, 27]. In brief, INFORM EGFR, HER2, and MET DNA Probe (Ventana Medical Systems, Inc.) and INFORM Chromosome 7 and 17 Probe (Ventana Medical Systems, Inc.) were visualized on the same slides according to the manufacturer's protocols. EBER-ISH was performed in all cases as previously described [42]. As previously described, cases showing diffuse strong positivity in the nuclei of all tumor cells were defined as EBER-ISH-positive [43].

SISH slides were reviewed by two individual pathologists (C. K. Park and H. Kim) and RTK gene signals were counted in at least 60 tumor cells per TMA core using a light microscope with ×40 objective. In some tumor cells, clusters of signals showing many copies of RTK genes were identified. According to the interpretation guide for Ventana INFORM HER2 DNA probe (Ventana Medical Systems, Inc.), small clusters were counted as 6 signals and large clusters as 12 signals. Gene-to-chromosome ratio and copy numbers per nuclei of each RTK gene were calculated. A gene-to-chromosome ratio greater than 2.0 was considered amplification. Cases showing amplification of any RTK genes in SISH were classified as RA-GCs.

### Subgrouping of AGCs

All AGCs were divided into four subgroups using two classification systems. First, all cases were classified by *putative molecular classification* based on findings of the TCGA study [14]. Each subgroup was defined as follows: (1) EBV-positive: EBER-ISH positive; (2) MMR-deficient: EBER-ISH negative and MMR-deficient; (3) pGS: EBER-ISH negative, MMR-proficient and p53 wild-pattern: and (4) pCIN: EBER-ISH negative, MMR-proficient and p53 mutant-pattern. In addition, we classified all cases into another four subgroups based on Lauren classification, MMR proteins and p53 IHC results, and EBER-ISH result. These four subgroups were defined as follows: (1) EBV-positive: EBER-ISH positive; (2) MMR-deficient: EBER-ISH negative and MMR-deficient; (3) D-pGS: EBER-ISH negative, MMR-proficient, p53 wild-type pattern, and diffuse type by Lauren classification; and (4) I-pCIN (p53 mutant-pattern): EBER-ISH negative, MMR-proficient, and p53 mutant-pattern with regardless of histologic type by Lauren classification, and I-pCIN (p53 wild-pattern): EBER-ISH negative, MMR-proficient, p53 wild-pattern and intestinal or mixed type by Lauren classification. We designated this system *histologic-molecular classification*.

### Statistical analysis

Statistical calculation was performed with SPSS version 21.0 (IBM, Chicago, IL, USA). Cross-table analysis (chi-square test) or Fisher's exact test was used to evaluate the relationship between IHC/SISH results and variable clinicopathologic factors. For the comparison of patient age, the student's *t* test was used. RFS and OS were estimated by the Kaplan-Meier method with log-rank test. Multivariate regression was analyzed using the Cox proportional hazards model. Significance statements refer to *P*-values of two-tailed tests < 0.05.

## SUPPLEMENTARY FIGURES AND TABLES


