# Supplementary material for: Receptor tyrosine kinase amplified gastric cancer: Clinicopathologic characteristics and proposed screening algorithm
Source: Oncotarget. 2016 Sep 27;7(44):72099–112. doi: 10.18632/oncotarget.12291 (PMC5342148; doi:10.18632/oncotarget.12291)
Supplement: Supplementary file 1 [file oncotarget-07-72099-s001.pdf]

Receptor tyrosine kinase amplified gastric cancer: Clinicopathologic characteristics and proposed screening algorithm

SUPPLEMENTARY TABLE AND FIGURES

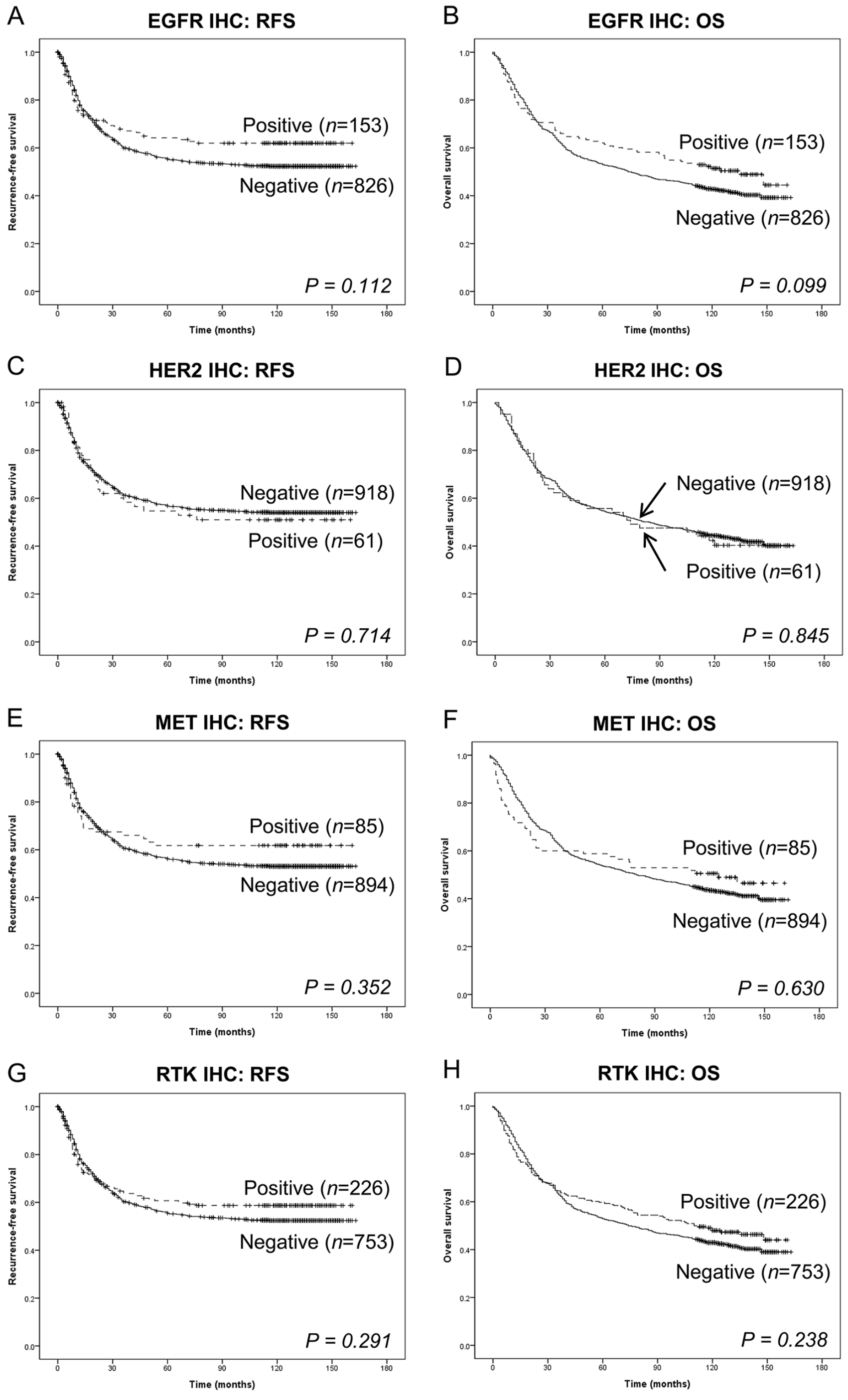

Supplementary Figure S1: Recurrence-free survival and overall survival according to RTK IHC results. No significant difference was observed for recurrence-free survival and overall survival based on expression status of EGFR **A** and **B.**, HER2 **C** and **D.**, or MET **E** and **F.** Also, positivity for any RTKs revealed no significant differences in recurrence-free survival and overall survival (G and H).

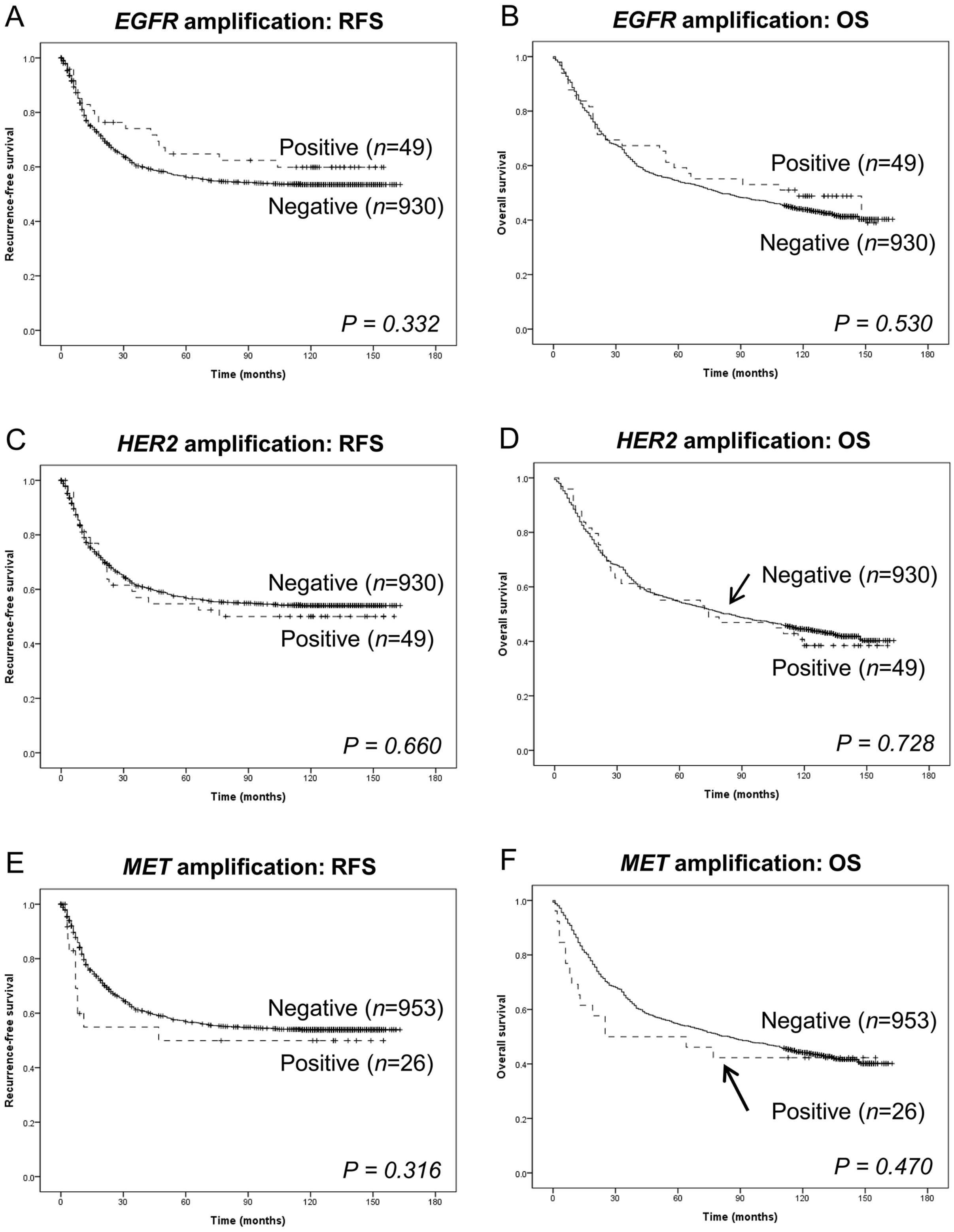

Supplementary Figure S2: Recurrence-free survival and overall survival according to RTK gene amplification status. No significant difference was observed for recurrence-free survival and overall survival according to EGFR **A** and **B.**, HER2 **C** and **D.**, and MET **E** and **F.** gene amplification status.

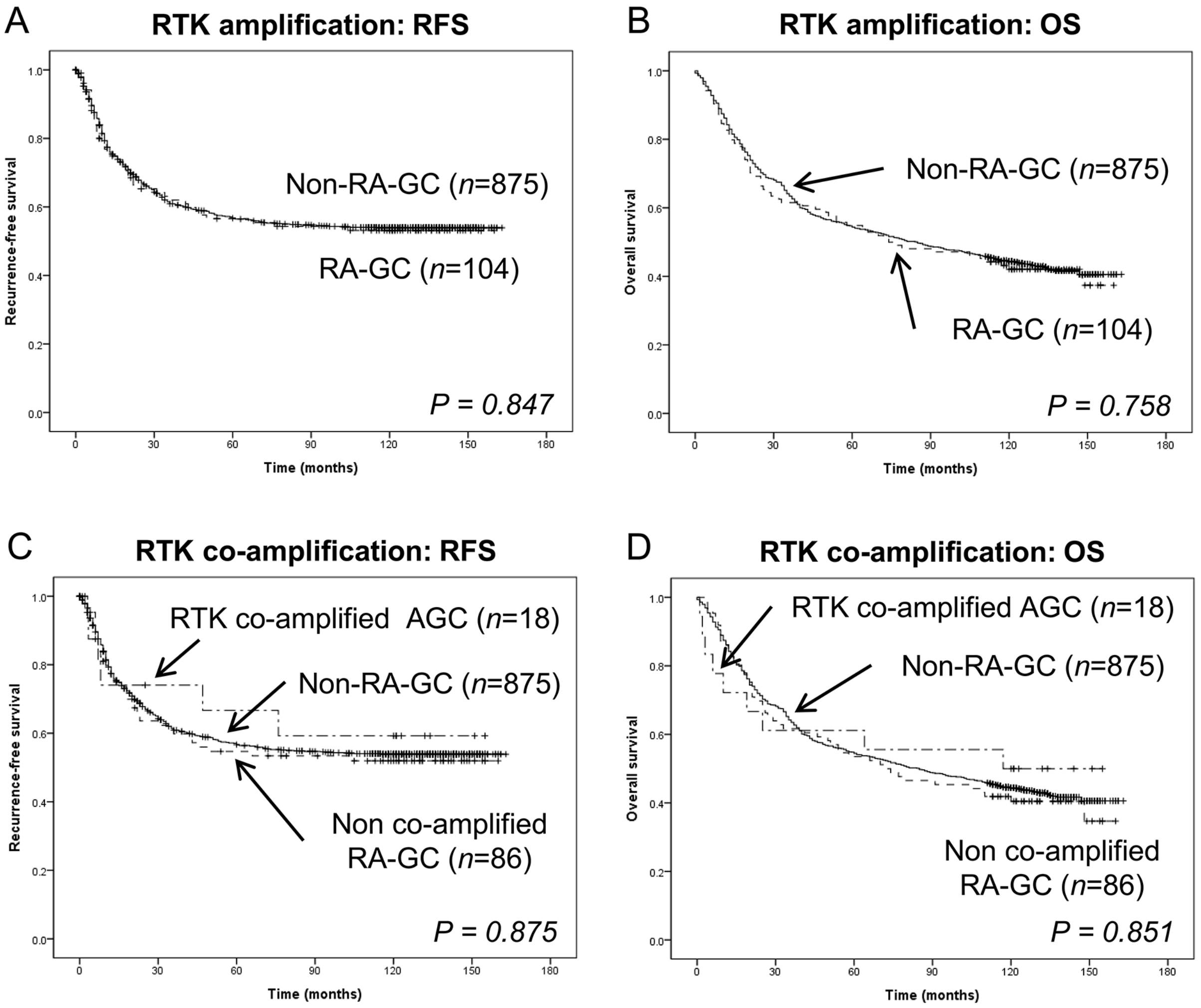

Supplementary Figure S3: Recurrence-free survival and overall survival based on RTK gene co-amplification status. No significant difference was observed for recurrence-free survival and overall survival between RA-GCs and non-RA-GCs **A** and **B.** In analysis according to the RTK gene co-amplification status, no significant differences were observed among non-RA-GCs, non co-amplified RA-GCs, and co-amplified RA-GCs **C** and **D.**

Supplementary Table S1: Clinicopathologic characteristics of 104 RA-GCs according to RTK gene co-amplification status

Category	Variables	No. of cases(n = 104)	RTK gene co-amplification				*P*-value
			Positive (%) (n = 18)		Negative (%) (n = 86)		
Age (years)			62.2 ± 7.97		59.8 ± 10.0		0.280
Sex	Male	76	12	(66.7)	64	(74.4)	0.562
	Female	28	6	(33.3)	22	(25.6)	
Location	Lower third	66	13	(72.2)	53	(61.6)	0.436
	Upper and mid-third	38	5	(27.8)	33	(38.4)	
Size	≤ 5 cm	52	12	(66.7)	40	(46.5)	0.194
	> 5 cm	52	6	(33.3)	46	(53.5)	
Differentiation	Differentiated	44	6	(33.3)	38	(44.2)	0.443
	Undifferentiated	60	12	(66.7)	48	(55.8)	
Lauren classification	Intestinal or mixed	90	15	(83.3)	75	(87.2)	0.706
	Diffuse	14	3	(16.7)	11	(12.8)	
LVI	Absent	64	10	(55.6)	54	(62.8)	0.601
	Present	40	8	(44.4)	32	(37.2)	
LNM	Absent	64	5	(27.8)	21	(24.4)	0.769
	Present	40	13	(72.2)	65	(75.6)	
Pathologic T stage	T2	19	3	(16.7)	16	(18.6)	0.848
	T3	45	7	(38.9)	38	(44.2)	
	T4	40	8	(44.4)	32	(37.2)	
p53 IHC	Wild-type pattern	22	2	(11.1)	20	(23.3)	0.350
	Mutant pattern	82	16	(88.9)	66	(76.7)	
EBER-ISH*	Negative	100	17	(100)	83	(97.6)	0.523
	Positive	2			2	(2.4)	
MMR protein IHC**	MMR-proficient	96	17	(94.4)	79	(92.9)	0.818
	MMR-deficient	7	1	(5.6)	6	(7.1)	
Overall stage	II	13	2	(11.1)	11	(12.8)	0.914
	III	25	5	(27.8)	20	(23.3)	
	IV	66	11	(61.1)	55	(64.0)	

LVI: lymphovascular invasion; LNM: lymph node metastasis; IHC: immunohistochemistry; EBV-ISH: Epstein-Barr virus *in situ* hybridization; MMR protein: mismatch repair gene related protein
* Evaluated in 102 cases
** Evaluated in 103 cases
